# Activation of the autophagy pathway by Torovirus infection is irrelevant for virus replication

**DOI:** 10.1371/journal.pone.0219428

**Published:** 2019-07-15

**Authors:** Ginés Ávila-Pérez, Elisabet Diaz-Beneitez, Liliana L. Cubas-Gaona, Gliselle Nieves-Molina, Juan Ramón Rodríguez, José F. Rodríguez, Dolores Rodríguez

**Affiliations:** 1 Department of Molecular and Cellular Biology, Centro Nacional de Biotecnología, CSIC, C/Darwin, Madrid, Spain; 2 Centro de Investigación en Salud Animal, INIA, Madrid, Spain; University of Padua, ITALY

## Abstract

Autophagy is a conserved eukaryotic process that mediates lysosomal degradation of cytoplasmic macromolecules and damaged organelles, also exerting an important role in the elimination of intracellular pathogens. Despite the antiviral role of autophagy, many studies suggest that some positive-stranded RNA viruses exploit this pathway to facilitate their own replication. In this study, we demonstrate that the equine torovirus Berne virus (BEV), the prototype member of the *Torovirus* genus (*Coronaviridae* Family, *Nidovirales* Order), induces autophagy at late times post-infection. Conversion of microtubule associated protein 1B light chain 3 (LC3) from cytosolic (LC3 I) to the membrane associated form (LC3 II), a canonical marker of autophagosome formation, is enhanced in BEV infected cells. However, neither autophagy induction, via starvation, nor pharmacological blockade significantly affect BEV replication. Similarly, BEV infection is not altered in autophagy deficient cells lacking either Beclin 1 or LC3B protein expression. Unexpectedly, the cargo receptor p62, a selective autophagy receptor, aggregates within the region where the BEV main protease (M^pro^) localizes. This finding, coupled with observation that BEV replication also induces ER stress at the time when selective autophagy is taking place, suggests that the autophagy pathway is activated in response to the hefty accumulation of virus-encoded polypeptides during the late phase of BEV infection.

## Introduction

Toroviruses have been described as enteric pathogens causing gastroenteritis and diarrhea in humans, especially in children, as well as in young domestic animals of high importance to the livestock industry, i.e. calves, piglets and foals [1–3]. They are enveloped, positive sense, single-stranded RNA viruses belonging to the *Nidovirales* order, which includes four families: *Arteriviridae*, *Roniviridae*, *Mesoviridae and Coronaviridae*, the latter containing two subfamilies, *Coronavirinae* (genera *Alpha-*, *Beta-*, *Delta-*, *and Gammacoronavirus*) and *Torovirinae (*genera *Bafinivirus* and *Torovirus*) [4]. The International Committee on Taxonomy of Viruses recognizes four torovirus species: human (HToV), bovine (BToV), porcine (PToV) and equine torovirus (EToV) [4]. The prototype member of the torovirus genus is the EToV strain Berne virus, the first identified torovirus (BEV), isolated from a diarrheic horse [5]. BEV was first adapted to cell culture, and for this reason, it is the best characterized torovirus. Nevertheless, other cell-culture adapted BToV strains are also currently available [6–8].

The torovirus genome consists of a single RNA molecule (ca. 27 kb) displaying an organization similar to that of other nidoviruses [9–13]. The first two-thirds of the genome carry two large overlapping open reading frames, ORF1a and ORF1b, directly translated from the viral genome, rendering two large polyproteins (pp1a and pp1ab), which are proteolytically processed by virus-encoded proteases (the papain-like proteinase [PLP] and the main proteinase [M^pro^]) to release replication and transcriptional machinery virus components (e.g. the RNA-dependent RNA polymerase [RdRp] and the helicase [Hel] amongst others) [1, 10, 14, 15]. The rest of the viral genome harbors ORFs encoding structural proteins: spike (S) [16, 17], membrane (M) [18, 19], hemagglutinin-esterase (HE) [20–22], and nucleocapsid (N) [14, 23], which are individually expressed from a nested set of 3’-coterminal subgenomic mRNAs [11, 24, 25].

Accumulating evidence from different positive-strand RNA viruses indicates that in this heterogeneous group of viruses, genome replication and transcription invariably occur in close association with highly modified cellular membranes of diverse origins [26–28]. In fact, the presence of double membrane vesicles (DMVs) in the cytoplasm of cells infected with different nidoviruses has been associated with the formation of their replication and transcription complexes (RTCs) [29–35]. In this regard, we recently described the presence of DMVs in the cytoplasm of BEV-infected cells, providing new evidence supporting the notion that nidoviruses share a common replicative structure based on DMV arranged clusters [15]. However, the mechanism and host factors required for the generation of these structures remain unknown. Because DMV morphology is akin to that of autophagosomes, and given that several pathogens have been shown to hijack the autophagy machinery, it has been postulated that some positive-strand RNA viruses may exploit the autophagy machinery to induce DMV formation [36–39].

Autophagy is a conserved eukaryotic mechanism mediating the removal of long-lived cytoplasmic macromolecules and damaged organelles via a lysosomal degradation pathway to maintain cellular homeostasis. During autophagy, cytoplasmic material is engulfed by double-membraned vesicles, termed autophagosomes, which are later fused with lysosomes for the degradation of enclosed materials by lysosomal hydrolases [40, 41]. Many autophagy-related genes (Atg) are involved in the formation and regulation of the autophagy pathway [42]. The ULK1-Atg13-FIP200 class III phosphatidylinositol 3-kinase complex (PI3K), which contains Beclin 1 and vps34, regulates the initiation and nucleation of autophagosome membranes [43]. Furthermore, two ubiquitin-like conjugation systems, i.e. Atg12 and Atg8/LC3, are involved in the elongation and closure of the autophagosome, converting the cytosolic form of the microtubule associated protein 1B light chain 3 (LC3 I) into the membrane associated type (LC3 II) [44]. Mammalian cells contain three LC3 isoforms referred to as A, B and C, being LC3B the most widely used.

Several studies have reported that members of the *Nidovirales* order trigger autophagy in host cells. However, the specific role of this pathway in nidovirus replication remains controversial [45–55]. As summarized by Cong and coworkers [55], autophagy may play opposite effects, either promoting virus replication or exerting an antiviral role, depending on the virus under analysis. In addition, an unconventional use of LC3 has been associated with mouse hepatitis virus (MHV) and equine arteritis virus (EAV), members of the *Coronaviridae* and *Arteriviridae* families, respectively. Although canonical autophagy is not required for the replication of these two viruses, the non-lipidated LC3 I form is essential for their replication [47, 53]. However, as yet, nothing is known about the potential interplay between members of the *Torovirus* genus and autophagy.

In this study, we provide evidence that autophagy is induced in BEV-infected cells by monitoring both autophagy signaling proteins and the autophagic flux. Furthermore, using pharmacological agents and RNA interference to modulate the autophagy pathway, we have analyzed the effect of the autophagy machinery on the BEV life cycle. Although BEV infection triggers an autophagy response, our results clearly indicate that this cellular pathway is irrelevant for BEV replication in tissue culture.

## Materials and methods

### Cells and viruses

Equine dermal (E. Derm) cells (NBL-6; ATCC CCL-57), human fetal lung fibroblasts (MRC-5) (ATTC CCL-171) and HEK 293T/17 cells (ATCC CRL-11268) were cultured in Dulbecco’s modified Eagle’s medium (DMEM) (Gibco) supplemented with non-essential amino acids (1%), gentamicin (50 μg/mL), penicillin (100 IU/mL), streptomycin (100 μg/mL) (Sigma-Aldrich), fungizone (0.5 μg/mL) (Gibco) and 15% of foetal calf serum (FCS) for E. Derm cells or 10% for the rest of the cells. The equine torovirus, strain Berne P138/72 (BEV), was used to infect E. Derm and MRC-5 cell monolayers as described previously [56]. A multiplicity of infection of 2.5 plaque-forming units per cell (pfu/cell) was used in all experiments. BEV inactivation was achieved by UV-irradiation as described previously [56].

### Reagents and antibodies

Rabbit polyclonal (L75423) and mouse monoclonal (LC3B-6) antibodies against LC3B were purchased from Sigma-Aldrich. Rabbit polyclonal antibodies against p62 (H-240) and Beclin 1 (H-300), as well as, a mouse monoclonal antibody against actin (C-4) were purchased from Santa Cruz Biotechnology. An additional rabbit polyclonal antibody against p62 was purchased from Cell Signaling (#5114). Rabbit polyclonal antibodies specific for N, M and RdRp BEV proteins, as well as a rat polyclonal antibody specific for M^pro^ were previously described [15, 19, 56]. Horseradish peroxidase-conjugated and Alexa Fluor-conjugated secondary antibodies were purchased from Sigma-Aldrich and Invitrogen, respectively.

3-Methyladenine (10 mM; 3-MA), wortmannin (5 μM; Wn), Earle´s balanced salt solution (EBSS), ammonium chloride (20 mM; NH_4_CL), thapsigargin (200 nM; TG) and dithiothreitol (2 mM; DTT) were purchased from Sigma-Aldrich. Hydroxychloroquine (17 μM; HCQ) was purchased from Sanofi-Synthelabo. Epoxomicin (200 nM; Epox) was purchased from Peptanova.

### Plasmids

The pEGFP-LC3 plasmid (Addgene plasmid #21073) was a gift from Dr. Tamotsu Yoshimori [44]. The pDest-mCherry-EGFP-LC3B plasmid was kindly provided by Dr. Terje Johansen [57]. Lentiviral pLKO.1-puro plasmids expressing MISSION short hairpin RNA (shRNA) against Beclin 1 (TRCN0000033549) and LC3B (TRCN0000155417 (shRNA LC3B 1) and TRCN0000153286 (shRNA LC3B 4)) were purchased from Sigma-Aldrich. The lentiviral pLKO.1-puro plasmid expressing a scrambled shRNA (shRNA control) was kindly provided for Dr. Pablo Gastaminza [58]. Lentiviral packaging plasmids pMDLg/pRRE (Addgene plasmid #12251), pM2.G (Addgene plasmid #12259) and pRSV-Rev (Addgene plasmid #12253) were a gift from Dr. Didier Trono [59]. The retroviral pSuper.retro.puro vector containing a shRNA sequence for Beclin 1 was kindly provided for Dr. William Maltese [60]. A similar retroviral vector containing a scrambled shRNA sequence (5´-CTTGTTCGTTGGTAACTACATTCAAGAGATGTAGTTACCA-ACGAACAA-3´) was generated as described [61]. The retroviral packaging plasmid pCL-Ampho was purchased from Novus Biologicals.

### SDS-PAGE and Western blot

Cells were lysed at the indicated times pi directly in Laemmli’s loading buffer supplemented with 5% β-mercaptoethanol. Protein lysates were subjected to SDS-PAGE and transferred to nitrocellulose membranes (Bio-Rad). Membranes were blocked for 1 h in Tris-buffered saline supplemented with 0.05% Tween 20 (TBS-T) containing 5% non-fat dry milk, and later incubated with primary antibodies diluted in the same buffer at 4°C overnight. Then, membranes were washed with TBS-T, incubated with appropriate dilutions of corresponding horseradish peroxidase-labeled secondary antibodies for 1 h at room temperature and washed again. Detection of immunoreactive proteins was carried out using the enhanced chemiluminescence Western blot detection kit (ECL, GE Health Care). Non-saturated films were scanned and relative protein values quantified by gel densitometry using the ImageJ software according to Luke Miller’s instructions available in http://lukemiller.org/index.php/2010/11/analyzing-gels-and-western-blots-with-image-j/. Alternatively, Western blots were imaged using a ChemiDoc^TM^Touch Imagin System (BioRad), and relative protein band intensities determined using the Image Lab Touch Software 2.4 provided with the equipment.

### Quantitative RT-PCR

E. Derm cells infected with BEV were harvested at the indicated times pi, washed three times with PBS and processed for RNA extraction with the RNeasy Mini kit (Quiagen) following the manufacturer’s instructions. Reverse transcription (RT) reaction was performed with 500 ng of RNA using a random hexamer mixture (Roche Applied Science) as primers and SuperScript III reverse transcriptase (Invitrogen). cDNA amplification was performed on an ABI Prism 7000 sequence detection system (Applied Biosystems) using Power SYBR Green Master Mix (Invitrogen). To detect the viral RNA, an oligonucleotide primer pair was designed based on the BEV N coding gene (N_F_: 5’-ACAGCGTGACCCAGCTTTTC-3’ and N_R_: 5’- TTTGACGGCTGCGATTCTG-3’). The housekeeping gene hypoxanthine phosphoribosyltransferase 1 (HPRT1) was used for normalization (HPRT1_F_: 5’-TGACACTGGCAAAACAATGCA-3’ and HPRT1_R_: 5’-GGTCCTTTTCACCAGCAAGCT-3’). Dilutions of plasmids containing the sequence amplified by each set of primers run in parallel were used to establish the corresponding standard curves.

### Virus titration

Supernatants of BEV-infected E. Derm cells were collected and the production of infectious extracellular virus was determined by plaque assay. Briefly, confluent monolayers of E. Derm cells were infected with ten-fold dilutions of the BEV-containing supernatants. After virus adsorption, cell monolayers were overlaid with a 1:1 mixture of 2xDMEM and 1.9% agar, containing 2% FCS and 0.05 mg/ml of DEAE-dextran. At 72 h pi, the agar overlay was removed and monolayers stained with a 1% crystal violet solution in 2% methanol to determine the number of individual virus-induced lysis plaques.

### Immunofluorescence

At indicated times pi, cells grown on glass coverslips were fixed with 4% paraformaldehyde (PFA) in PBS and processed for immunofluorescence using a standard protocol. After washing with PBS, cells were permeabilized with 0.5% Triton X-100 in PBS, and then blocked with PBS containing 20% FCS. For immunofluorescence analysis with the anti-p62 antibody the cells were fixed with methanol at -20°C for 20 min and subsequently blocked with PBS containing 20% FCS. Thereafter, cells were incubated with primary antibodies diluted in PBS supplemented with 20% FCS in a humidified chamber for 1 h at 37°C. After this period, coverslips were washed with PBS and incubated with secondary antibodies conjugated to either Alexa Fluor 488 or 594, and DAPI for nuclei staining. Coverslips were mounted on microscope slides using ProLong Gold anti-fade reagent (Invitrogen). Images were acquired with a Leica TSC SP5 confocal laser scanning microscope with a step size of about 0.5 μm.

### Generation of E. Derm cells stably expressing GFP-LC3

E. Derm cells were transfected with the pGFP-LC3 plasmid using Lipofectamine 2000 (Invitrogen). At 48 h post-transfection, G418 (500 μg/ml; Sigma-Aldrich) was added to the culture medium. G418-resistant cell clones expressing GFP were isolated with cloning cylinders (Sigma-Aldrich) by using trypsin/EDTA, and then amplified by conventional culture methods. GFP-LC3 expression was analyzed in several clones by immunofluorescence and Western blot using anti-LC3 antibodies.

The number of cytosolic GFP-LC3 dots per cell was determined with the ImageJ software. Briefly, confocal images were acquired with a Leica TSC SP5, and the outline of the cells of interest was selected by the ImageJ ROI manager tool. Then, the GFP-LC3 dots in selected cells were counted using the “Analyze particles” tool using an automatic threshold Intermodes and a particle size of two pixels. The percentage of cells harboring GFP-LC3 dots was calculated considering only cells with 10 or more dots as positive ones. We determined a threshold of 10 dots per cell after concluding that the median of mock-infected cell samples was invariably below this number.

### Generation of E. Derm cells transiently expressing mCherry-GFP-LC3

E. Derm cells grown on coverslips (~2x10^5^ cells/well of MW24) were transfected with the pCherry-GFP-LC3 plasmid (500 ng/well) using Lipofectamine 2000 according to the manufacturer´s instructions. At 24 h post-transfection, cells were mock-treated, treated with the indicated drugs or infected with BEV. At the indicated times, cells were processed for immunofluorescence analysis as described above. Colocalization analyses were performed by determining the Pearson’s correlation coefficient using the JACoP tool in the Image J software [62].

### Production of retroviral particles and generation of Beclin 1 knockdown E. Derm cells

Retroviral particles were produced in 293T cells (~5x10^6^ cells/100 mm dish) by cotransfection of 4 μg of the retroviral vector pSuper.retro.puro, expressing a shRNA sequence for Beclin 1 (Beclin1-KD), or the control vector expressing a scrambled shRNA together with 4 μg of the packaging plasmid pCL-Ampho using Lipofectamine 2000. After 12 h, the transfection medium was replaced by fresh culture medium, and 24 h later supernatants containing replication-defective retrovirus were collected, filtered through a 0.45 μm filter and used to transduce E. Derm cells.

To generate stable Beclin 1 knockdown cells, E. Derm cells (~1.5x10^6^ cells/60 mm dish) were transduced with 3 ml of supernatant containing retrovirus together with 8 μg/ml of Polybrene (Sigma-Aldrich). This process was repeated twice, at 10 and 24 h post-transduction (pt), using 3 ml of supernatant and 4 μg/ml of Polybrene. At 32 h pt, the medium was replaced and cells maintained in fresh culture medium. At 48 h pt, puromycin was added to culture medium to reach a concentration of 2 μg/ml. After a 3–4 days selection period, cultures were maintained in regular medium supplemented with 0.5 μg/ml of puromycin.

### Production of lentiviral particles and generation of Beclin 1 knockdown E. Derm cells

Lentiviral particles were produced in 293T cells (~5x10^6^ cells/100 mm dish) by cotransfecting 10 μg of the lentiviral vector pKLO.1-puro plasmid expressing the shRNA against Beclin 1, or pLKO.1-puro plasmid expressing a scrambled shRNA (Control), together with packaging plasmids (10 μg of pMDLg/pRRE, 3.6 of μg pRSV-Rev and 5 μg of pM2.G) using a standard calcium phosphate transfection protocol [63]. 16 h later, the transfection medium was replaced by fresh culture medium. After 24 h, cell supernatants containing replication-defective lentiviruses were collected, filtered through a 0.45 μm filter and used to transduce E. Derm cells.

To generate a stable cell line, E. Derm cells were transduced with the smallest amount of supernatant sufficient to confer resistance to puromycin on 100% of the cells as described by Friesland and co-workers [58]. At about 12 h pt, puromycin (2 μg/ml) was added to the culture medium for the selection of transduced cells, and after 3–4 days the cells were passaged and maintained in regular medium supplemented with 0.5 μg/ml of puromycin.

### Production of lentiviral particles and generation of LC3B knockdown E. Derm cells

Lentiviral particles were produced in 293T cells by cotransfecting pKLO.1 LC3B plasmids with packaging plasmids as described above. Similarly, cells were selected with puromycin. In this case, cells were only maintained up to 5–12 days pt, and assays were performed during this time span.

### Determination of ER stress through the analysis of X-box binding protein 1 (Xbp1) mRNA splicing

E. Derm cells, either mock-infected or infected with BEV, were collected at different times pi and subjected to RNA extraction and RT reaction as described above. As control, RNAs were also extracted from cells treated for 20 minutes with 2 mM dithiotreitol (DTT, Sigma-Aldrich) or 200 nM thapsigargin (TC, Sigma-Aldrich). The resulting cDNAs were amplified by PCR using Platinum Taq DNA Polymerase (Invitrogen) and specific primers for *XBP1* amplification (i.e. XBP1_F_: 5’-CTGGAACAGCAAGTGGTAGA-3’ and XBP1_R_: 5’- CTGGGTCCTTCTGGGTAG-3’), as previously described [64]. Amplified DNA fragments were electrophoresed in 2% agarose gels in the presence of ethidium bromide (Invitrogen).

## Results

### BEV infection results in an increase of the LC3B II/LC3B I ratio

**Fig 1:**
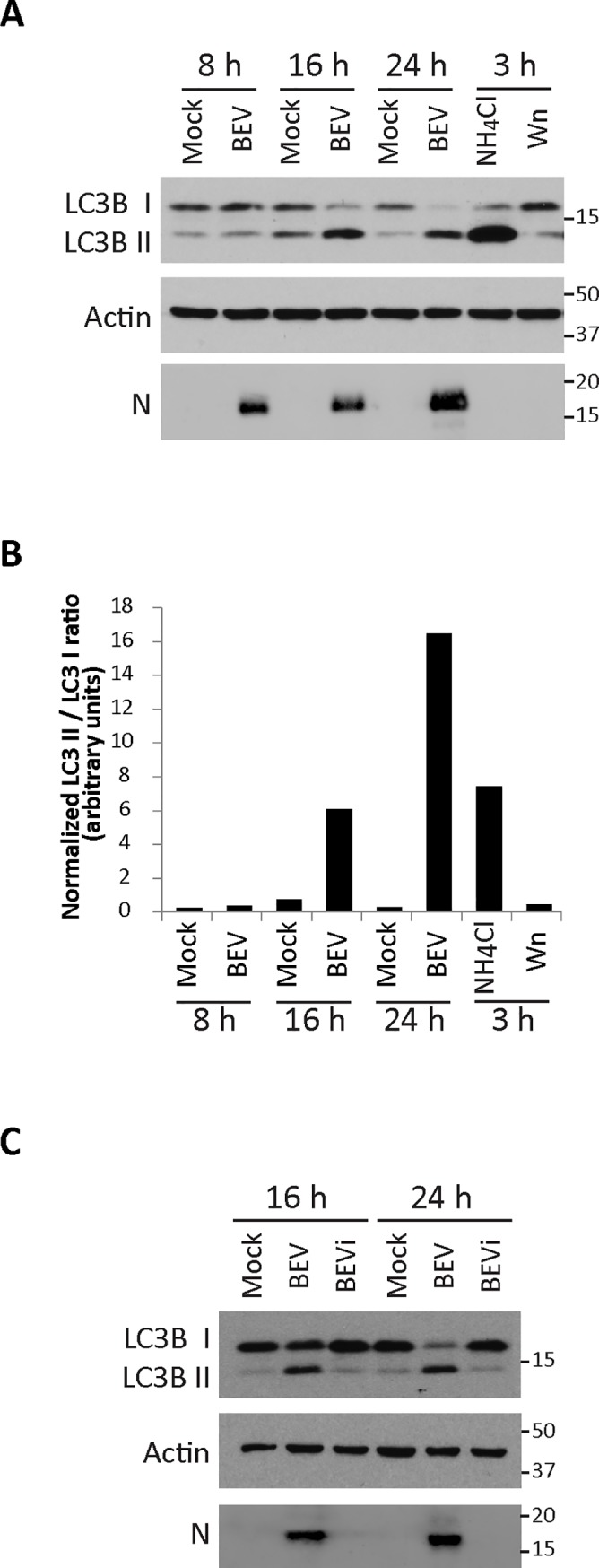
Analysis of LC3 lipidation in BEV infected cells. **A)** Western blot analysis of extracts of E. Derm cells, mock-infected or infected with BEV, collected at 8, 16 and 24 h pi. As control, extracts from E. Derm cells treated for 3 h with either ammonium chloride (20 mM, NH_4_CL) or wortmannin (5 μM, Wn) were included. A polyclonal anti-LC3B antibody was used to analyze the conversion of endogenous LC3B I to LC3B II. Anti-BEV N and anti-actin antibodies were used as infection and protein loading controls, respectively. **B)** Densitometric quantification of the LC3B II/LC3B I ratio (a representative example of three independent experiments is shown). Data were normalized using actin loading controls. **C)** Western blot analysis of extracts from E. Derm cells mock-infected, infected with BEV, or incubated with an equivalent amount of UV-inactivated BEV (BEVi), collected at 16 or 24 h pi. Western blots were performed with antibodies specifically recognizing LC3B, actin and the BEV N protein, respectively.

Some positive-stranded RNA viruses are capable of subverting the autophagy pathway for their benefit [65, 66]. To determine the possible effect that the BEV infection may exert on the autophagy pathway we followed the guidelines for monitoring autophagy [67]. First, we examined the lipidation status of endogenous LC3 protein in infected cells. For this, E. Derm cells were mock-infected or infected with BEV, lysed at 8, 16 or 24 h post-infection (pi) and the corresponding extracts used for Western blot analysis using a polyclonal anti-LC3B antibody. Newly synthesized BEV N protein was also analyzed to monitor virus infection. In addition, as control we used extracts from E. Derm cells treated during 3 h with ammonium chloride (NH_4_CL); a compound that impairs autophagosome to lysosome fusion, and thereby autophagosome degradation [68, 69]; or with wortmannin (Wn) that blocks autophagy by targeting PI3K [70]. As shown in Fig 1A, mock-infected cells showed a LC3B II level similar to that detected in LC3 lipidation deficient Wn-treated cells. In contrast, BEV infection resulted in an increased LC3B II/LC3B I ratio at 16 and 24 h pi, relative to uninfected cells. Similarly, the LC3B II/LC3 I ratio was altered in the NH_4_CL-treated cells, where the autophagosome-lysosome fusion was impaired. Densitometric analysis of corresponding protein bands confirmed the gradual increase of the LC3B II/LC3B I ratio along the infection (Fig 1B).

We also sought to determine whether the observed enhancement of the LC3B II/LC3B I ratio was dependent upon viral replication, or it was rather a direct consequence of virus entry. For this, E. Derm cells mock-infected, infected with BEV or incubated with an equivalent amount of UV-inactivated BEV (BEVi) were collected at 16 or 24 h pi, and analyzed by Western blotting. As expected, the BEV N polypeptide was not detected in BEVi-treated cells, indicating the absence of viral replication (Fig 1C). With regard to the LC3B protein, cells incubated with BEVi showed a LC3B lipidation akin to that observed in mock-infected cells, where most of the protein corresponds to the LC3B I form. However, as mentioned above, cells infected with BEV showed a significant increase in the relative LC3B II abundance when compared to that found in both mock-infected cells and BEVi-treated cells. Indeed, these results show that a BEV replication-competent virus is required to cause the accumulation of LC3B II.

### BEV induces the accumulation of autophagosomes in infected cells

**Fig 2:**
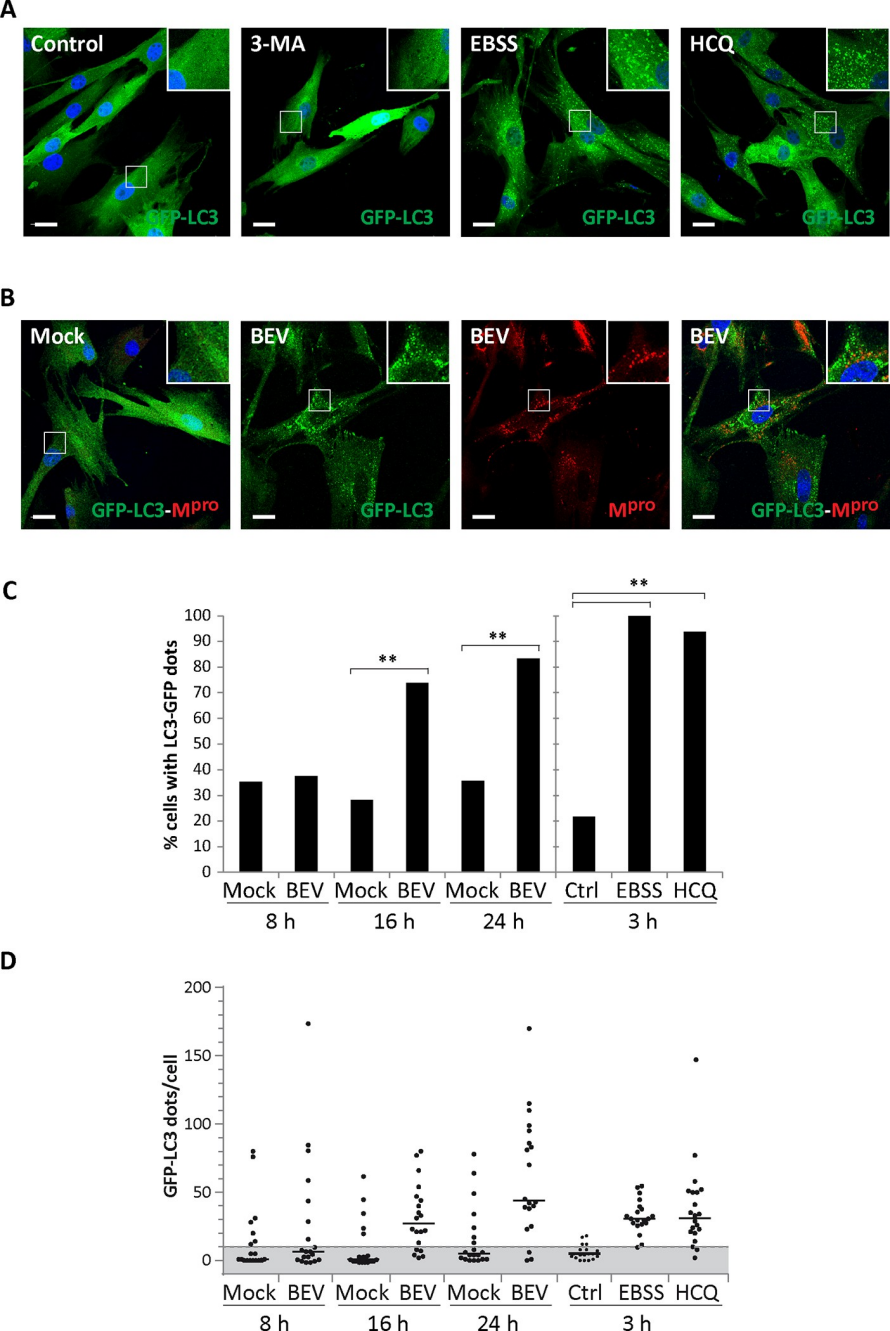
Redistribution of the GFP-LC3 protein in BEV infected cells. E. Derm_GFP-LC3 cells stably expressing a heterologous GFP-LC3 fusion protein were generated. **A)** E. Derm_GFP-LC3 cells were treated during 3 h with the autophagy inhibitor 3-MA (10 mM), with HCQ (17 μM), starved in EBSS medium or maintained in standard culture medium (Control). After treatment, cells were fixed and nuclei stained with DAPI. **B)** E. Derm_GFP-LC3 cells were mock-infected or infected with BEV, fixed at 16 h pi and immunolabeled with a specific antibody against the BEV protease M^pro^. Boxed areas correspond to representative fields shown at higher magnification. Scale bars, 25 μm. **C)** Quantification of cells harboring GFP-LC3 dots. E. Derm_GFP-LC3 cells, treated as in (B), were fixed at 8, 16 and 24 h pi, respectively, and used for GFP-LC3 dot quantification. As control, cells treated as in (A) were also quantified. Only cells with 10 or more GFP-LC3 dots were considered positive (n = 20). ** p<0.001. **D)** Number of GFP-LC3 dots per cell quantified in (C), where the medians are shown (horizontal bars). The threshold of 10 dots used in (C) is indicated in grey.

Another useful criterion to studying the autophagy pathway is the redistribution of intracellular LC3 from a diffuse localization (LC3 I) to a characteristic punctate cytoplasmic pattern (LC3 II) in autophagy vesicles [67]. To further analyze whether BEV infection affects the autophagy pathway, we generated E. Derm cells expressing a heterologous GFP-LC3 reporter protein. To assessing the correct redistribution of GFP-LC3 during autophagy, cells were treated for 3 h with the autophagy inhibitors 3-methyladenine (3-MA), that prevents autophagosome formation by inhibiting class III PI3K [71, 72], hydroxichoroquine (HCQ) specifically blocking lysosome-mediated proteolysis [73, 74], or with EBSS starvation medium to induce the autophagy pathway. Control cells were maintained in regular culture medium. As shown in Fig 2A, in cells where LC3 lipidation was impaired by 3-MA treatment, the GFP-LC3 polypeptide showed a diffuse cytoplasmic localization. However, after autophagy induction by starvation or autophagy flux blockade by the HCQ treatment, the GFP-LC3 protein reporter was distributed forming punctate structures. In control cells, the GFP-LC3 protein mostly showed a diffuse cytoplasmic localization as in 3-MA-treated cells. However, since the autophagic flux was not impaired, some punctate structures could also be observed in these cells.

Once the redistribution of GFP-LC3 under the different conditions was confirmed, cells were mock-infected or infected with BEV, fixed at 16 h pi and immunolabeled with the anti-M^pro^ antibody to monitor virus infection. As expected, while in mock-infected cells the GFP-LC3 reporter exhibited a dominant diffuse pattern, infected cells showed a clear punctate GFP-LC3 distribution (Fig 2B) akin to that observed in starved as well as in HCQ-treated cells (Fig 2A). Similar assays were subsequently performed to determine the percentage of the cell population harboring GFP-LC3 dots. As control, GFP-LC3 dots detected in untreated as well as in HCQ- and EBSS-treated cells were quantified. For this, cells were fixed at three time points, i.e. 8, 16 and 24 h pi, and only cells exhibiting 10 or more GFP-LC3 dots were considered positive (Fig 2C). This quantitative study showed a statistically significant increase in the percentage of infected cells bearing GFP-LC3 dots at 16 and 24 h pi when compared to that found in mock-infected cultures. However, no significant differences were detected at 8 h pi. Similarly, EBSS- and HCQ-treated cultures showed a significant increase in the percentage of positive cells compared to that found in untreated control monolayers. Although total GFP-LC3 dot numbers per cell were highly variable, this value was significantly higher in cells infected with BEV for 16 and 24 h, as well as in cells treated with EBSS or HCQ (Fig 2D).

Overall, these results suggest that the number of autophagosomes increases in BEV-infected cells at 16 and 24 h pi with respect to mock-infected cells. Although, this increase could be due to the activation of the autophagy pathway by BEV, at this point we cannot rule out the possibility that autophagosome degradation could be blocked by BEV infection, which would then promote their accumulation, as recently observed in Dengue virus infected cells [75].

### BEV does not block the autophagic flux

As mentioned above, an increase in the amount of autophagosomes can be due to either the induction of autophagy or the inhibition of the flux through autophagosome degradation. To check whether BEV infection induces autophagosome accumulation via inhibition of the autophagic flux, autophagosome acidification was monitored using a vector expressing the mCherry-GFP-LC3 fusion protein. The usefulness of this construct is based on the fact that GFP signal is quenched in acidic environments whereas the mCherry signal remains stable under these conditions. This allows monitoring autophagosome formation as well as the subsequent autophagosome-lysosome (autolysosome) fusion step.

**Fig 3:**
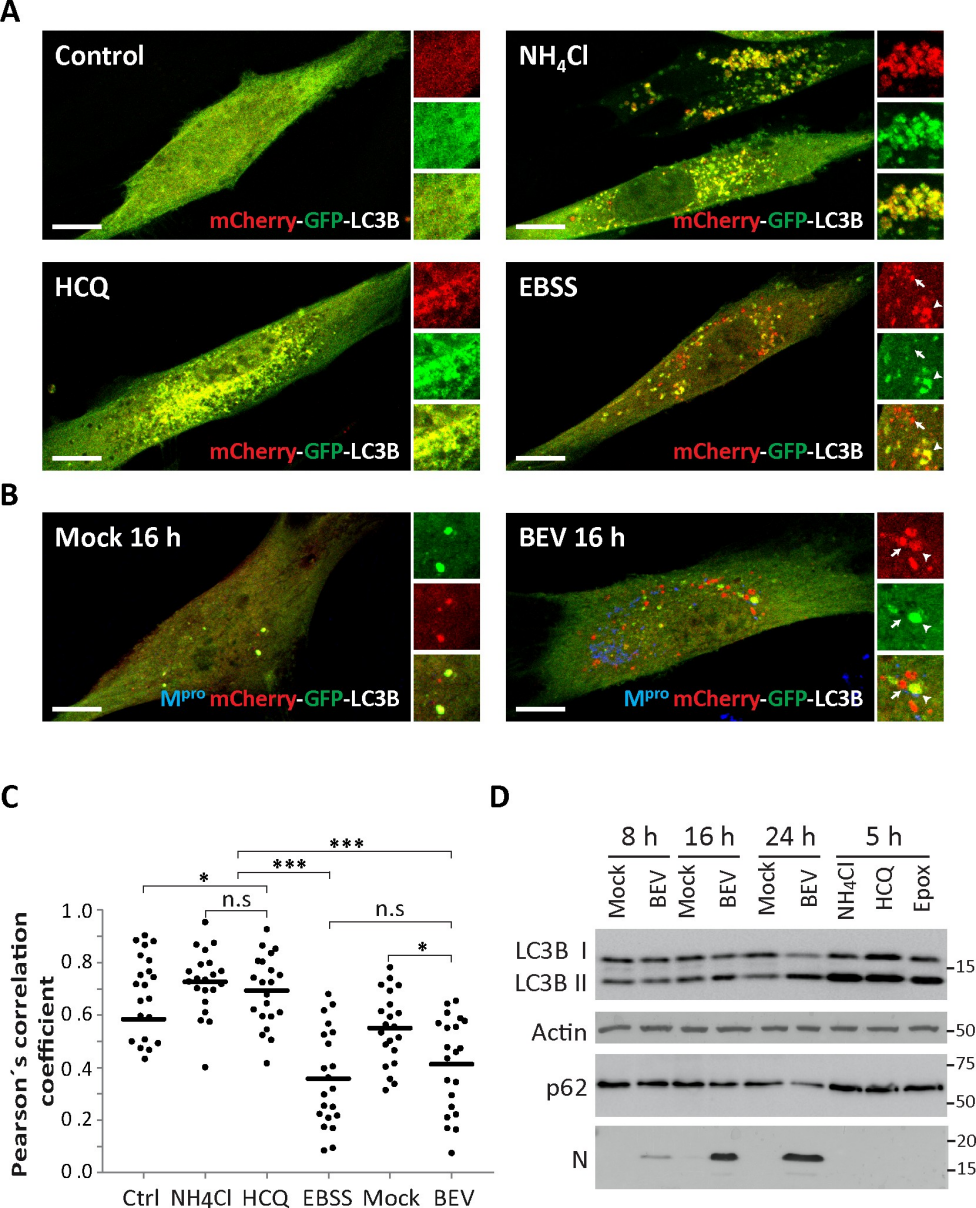
The autophagy flux during BEV infection. To check whether the infection with BEV induces autophagosome accumulation via inhibition of the autophagy flux, autophagosome acidification was monitored using a mCherry-GFP-LC3 expression vector. In an acidic environment the GFP signal (green) is quenched whereas the mCherry signal (red) remains stable, which allows us to monitor autolysosome formation following autophagosome-lysosome fusion (A-C). **A)** E. Derm cells were transfected with the mCherry-GFP-LC3 plasmid, and at 24 h post-transfection treated for 3 h with NH_4_CL (20 mM), HCQ (17 μM), starved with EBSS medium or left untreated. **B)** E. Derm cells transfected as in (A) were mock-infected or infected with BEV. At 16 h pi cells were fixed and processed for immunofluorescence using a specific antibody against the BEV M^pro^ protease. Each panel shows a general image. Right hand side small panels show enlarged images corresponding to representative cell cytoplasm areas displaying both GFP and mCherry signals (lower panels), the GFP (middle panels) or the mCherry signal (upper panels). Arrowheads and arrows indicate autophagosomes and autolysosomes, respectively. Scale bars, 10 μm. **C)** Pearson´s correlation coefficients between mCherry and GFP obtained from cells treated under the different conditions are shown (n = 20). Each dot represents one cell. Horizontal bars represent the mean. * p<0.05; *** p<0.001; n.s non-significant. **D)** To further determine whether the autophagy flux remains active during BEV infection, the turnover of cargo p62 protein was analyzed by Western blotting at different times pi. MRC5 cells, mock-infected or infected with BEV, were collected at 8, 16 and 24 h pi. As control, cells treated for 5 h with NH_4_Cl (20 mM), HCQ (17 μM) or with the proteasomal inhibitor epoxomicin (200 nM; Epox) were also analyzed. Western blot analysis was performed with antibodies against p62, LC3B and BEV N. Actin was used as a protein loading control.

In order to validate the assay, E. Derm cells were transfected with the mCherry-GFP-LC3 vector. At 24 h post-transfection cultures were treated for 3 h with the autophagy inhibitors NH_4_CL or HCQ, or maintained in either starvation (EBSS) or regular medium (Fig 3A). After the treatments, the mCherry-GFP-LC3 protein showed the expected patterns, i.e. from a diffuse localization in normal conditions (Control) to the characteristic punctate distribution in autophagy vesicles when autophagy is active. These vesicles can be non-acidic vesicles (autophagosomes), thus displaying mCherrry (red) and GFP (green) fluorescence, or acidic vesicles (autolysosomes) showing only red fluorescence. Under starvation conditions, when the flux is active, both types of vesicles were detected. However, when the autophagic flux was blocked by agents impairing lysosomal acidification (NH_4_CL or HCQ treatment) only neutral structures, simultaneously exhibiting both red and green fluorescence, were found. To analyze whether the autophagic flux is inhibited during BEV infection, E. Derm cells were transfected with the mCherry-GFP-LC3 vector. At 24 h post-transfection cultures were mock-infected or infected with BEV. At 16 h pi the cells were fixed and processed for immunofluorescence analysis using a specific antibody against the BEV M^pro^ protease. As observed in the Fig 3B, cells infected with BEV displayed an active flux, akin to that found in cells subjected to starvation with EBSS, harboring both acidic and neutral vesicles. To confirm these observations, mCherry/GFP colocalization was assessed by determining Pearson´s correlation coefficients (r) (Fig 3C). As expected, high correlation values (r≥0.5) were obtained when the autophagic flux was blocked by NH_4_CL or HCQ, with an r average of 0.7 ± 0.13 and 0.73± 0.12, respectively. However, when autophagy flux was active under starvation conditions (EBSS), a lower correlation mCherry/GFP was detected, with an r average of 0.36± 0.18. Significantly, a similar situation was observed with BEV-infected cells, showing an r average of 0.41± 0.18. Despite the r value cell to cell fluctuations, there were no statistically significant differences between EBSS-treated and BEV-infected cells. In contrast, NH_4_CL and HCQ-treated cells showed significant differences when compared to both BEV-infected and EBSS-treated cells.

Assessment of p62 (also called sequestosome 1 or SQSTM1) cargo receptor protein turnover can also be used to determine autophagy flux, where the degradation of p62 indicates an active flux [67]. To further confirm that the autophagic flux remains active in BEV-infected cells, we analyzed the fate of the p62 protein during the course of infection (Fig 3D). Unfortunately, none of the tested anti-p62 antibodies recognize the equine p62 protein from E. Derm cells. Therefore, these experiments were performed using human MRC5 cells, which we have previously described to be susceptible to BEV infection (44). MRC5 cells mock-infected or infected with BEV were collected at 8, 16, and 24 h p.i. In addition, mock-infected cells treated for 5 h with NH_4_Cl (20 mM) and HCQ (17 μM) or with the proteasomal inhibitor epoxomicin (200 nM) were used as controls. First, to determine whether the autophagy pathway was also altered in BEV-infected MRC5 cells, cell extracts were analyzed by Western blotting with antibodies against LC3. As shown in Fig 3D, conversion of LC3 I to LC3 II was detected at 16 and 24 h pi. Virus infection was monitored by Western blot using antibodies against the BEV N polypeptide. As expected, a gradual increase of the relative N protein accumulation was observed during the course of infection. Significantly, a clear reduction in the p62 relative intensity, as compared with that of mock-infected cells, was observed at 16 and 24 h pi (38% and 51% respectively). By contrast, an increased p62 accumulation was observed in drug-treated cells. These results clearly demonstrate that BEV infection also induces autophagy in this human cell line, and that it does not block the autophagy flux.

### BEV infection is not affected by the modulation of the autophagy pathway

**Fig 4:**
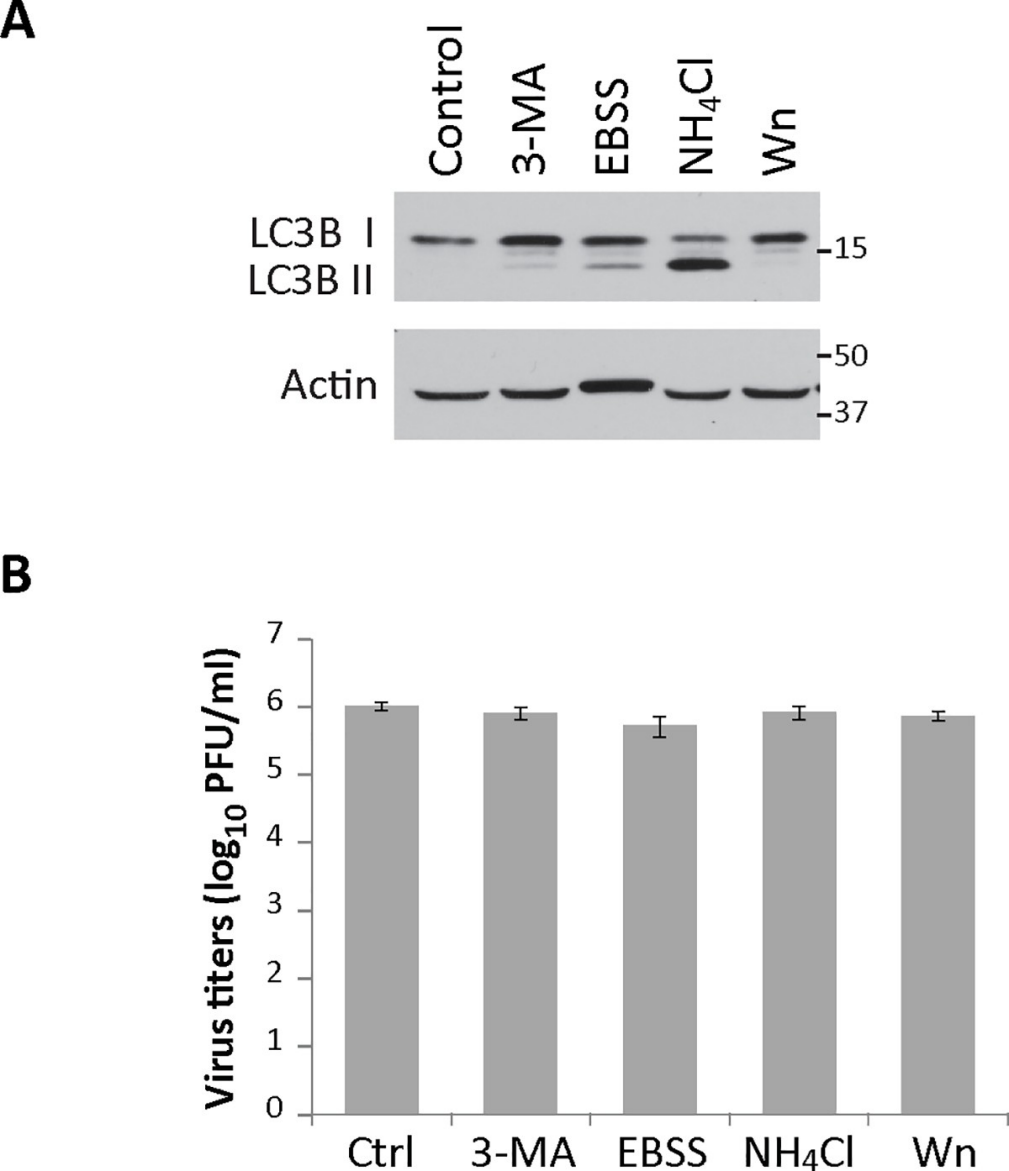
Effects of pharmacological modulation of the autophagic pathway on BEV infection. To analyze the role of autophagy on BEV infection, E. Derm cells were pretreated during 3 h with 3-MA (10 mM), EBSS, NH_4_CL (20 mM), Wn (5 μm) or maintained in regular medium. Cultures were subsequently infected with BEV and maintained thereafter in regular medium without drugs. **A)** E. Derm cells pretreated during 3 h were lysed and analyzed by Western blot before being infected with BEV. The conversion of LC3B I to LC3B II was analyzed using an anti-LC3B polyclonal antibody. Actin was used as loading control. **B)** At 16 h pi, supernatants from infected cells (n = 3) were collected and used to determine extracellular viral yields.

In order to evaluate the impact of the autophagy pathway on BEV replication, we first analyzed the effect that the pharmacological modulation of this pathway might exert on BEV infection. For this, E. Derm cells were pretreated during 3 h with 3-MA, EBSS, NH_4_CL or Wn. Thereafter, cultures were infected with BEV and maintained in regular medium without inhibitors for 16 h. As control, some cultures were maintained in regular culture medium throughout the whole experiment. Before the infection, cell extracts were analyzed by Western blot to check for the effectiveness of the different treatments on autophagy modulation (Fig 4A). As expected, LC3B lipidation was impaired following treatment with 3-MA or Wn, and consequently LC3B II levels were negligible, as in control cells. However, in cells where autophagosome degradation was prevented (NH_4_CL), the LC3B protein was largely found as LC3B II. In EBSS-starved cells the autophagic flux remains active, thus allowing simultaneous autophagosome formation and degradation. Accordingly, a relatively low LC3B II proportion was observed. Under these conditions, cells were infected with BEV and the corresponding supernatants collected at 16 h pi. These extracts were used to quantify extracellular virus production. As observed in Fig 4B, no significant variations on extracellular BEV yields were detected following any of the described pharmacological treatments. To further assess the validity of these results, a similar experiment was performed by treating cultures with the proteasome inhibitor MG132 previously established to inhibit BEV replication. As expected, a reduction exceeding one log_10_ unit in extracellular virus titers was attained (Data not shown).

**Fig 5:**
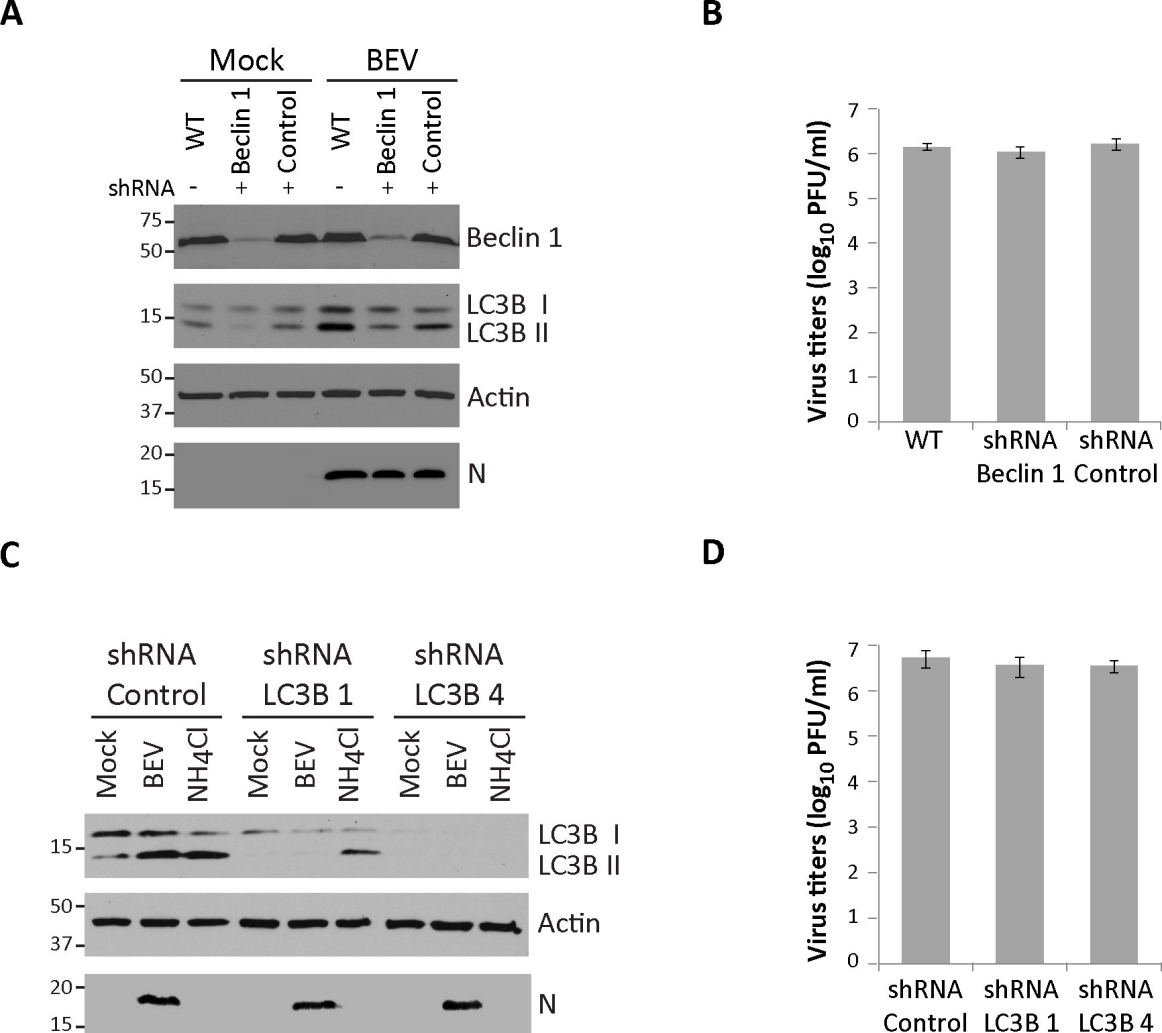
Effect of knockdown of key autophagy proteins on BEV infection. **A)** E. Derm cells were transduced with lentiviral vectors expressing short harping RNA against Beclin 1 (shRNA Beclin 1) or an irrelevant scrambled shRNA (shRNA Control). E. Derm cells (WT), E. Derm shRNA Beclin 1 and E. Derm shRNA control cells were mock-infected or infected with BEV. Cells were lysed at 16 h pi, and the corresponding extracts analyzed by Western blotting using antibodies recognizing Beclin 1, LC3 or the BEV N protein. The anti-actin antibody was used as loading control. **B)** Supernatants produced in (A) were collected (n = 3) and used to determine extracellular viral production. **C)** E. Derm cells were transduced with lentiviral vectors expressing shRNA against LC3B (shRNA LC3B 1 and shRNA LC3B 4) or an irrelevant shRNA (shRNA Control). Cells were mock-infected or infected with BEV, harvested at 16 h pi and analyzed by Western blot with antibodies directed against LC3B, BEV N or actin. As control, cells were treated for 3 h with NH_4_CL (20 mM). **D)** Cell culture supernatants produced in (C) were subjected to virus titration as in (B).

In view of results gathered with pharmacological agents, we extended our study by using specific short hairpin RNAs (shRNA) targeting two key players in the autophagy pathway. First, Beclin 1 deficient cells were generated by transducing E. Derm cells with a lentiviral vector expressing a shRNA against Beclin 1 mRNA. Similarly, shRNA control cells were generated by transducing E. Derm cells with a lentiviral vector expressing a scrambled shRNA sequence. Cells transduced with Beclin 1 or control shRNA as well as untransduced E. Derm cells (WT), were mock infected or infected with BEV, lysed at 16 h pi and analyzed by Western blot using antibodies against Beclin 1 and LC3B. An antibody specifically recognizing the BEV N protein was also used to monitor virus infection. As observed in Fig 5A, the amount of Beclin 1 was strongly diminished in Beclin 1 deficient cells, exhibiting a reduction of about 80% compared with the control cells which remained unaltered during BEV infection. As expected, LC3B I to LC3B II conversion decreased in Beclin 1 deficient cells, showing a LC3B II level in infected cells similar to those detected in mock-infected WT or control cells, yet slightly higher than that found in mock-infected Beclin 1 deficient cells. The remaining expression (~20%) of Beclin 1 in lentivirus transduced cells could account for presence of the minor LC3B II band observed in infected cells. Significantly, under these conditions the expression of the N viral protein was similar to that detected in WT and control BEV-infected cells. To further assess the effect of Beclin 1 silencing on BEV infection, extracellular viral yields were determined using infected cell supernatants collected at 16 h pi. As shown in Fig 5B, no significant changes in the production of infectious BEV were detected when comparing autophagy deficient cells, lacking Beclin 1, and control cells. Similar results were obtained using E. Derm cells transduced with another retroviral vector expressing a different shRNA against Beclin 1 (S1 Fig).

Data gathered with pharmacological agents as well as with the silencing of a key autophagy protein such as Beclin 1, suggest that classic autophagy is not required for BEV infection. Nevertheless, an autophagy-independent LC3 function on virus replication has been described in some nidoviruses [47, 53]. To determine whether the LC3 protein might also exert an autophagy independent role on the BEV life cycle, LC3B deficient cells were generated. For this, E. Derm cells were transduced with lentiviral vectors expressing shRNAs against LC3B (shRNA LC3B 1 or shRNA LC3B 4) or a scrambled shRNA sequence. Cells transduced with LC3B or control shRNAs were mock-infected or infected with BEV, lysed at 16 h pi and analyzed by Western blot with antibodies specific for LC3B and BEV N polypeptides. As control, cells in which autophagosome degradation was impaired by NH_4_CL treatment for 3 h were also included in the experiments. The relative amount of LC3B was significantly diminished in LC3B deficient cell lines, with a reduction of about 80–95% in the total amount of protein compared with control cells (Fig 4C). Like in previous assays with Beclin 1, the amount of viral protein accumulated during BEV infection was similar to that obtained in infected control cells. In addition, supernatants from infected cells were collected at 16 h pi to determine virus yields. Again, significant changes between LC3B deficient cells and control cells were not detected (Fig 5D).

Overall, these results show that although BEV induces the autophagy pathway, either positive or negative modulation of this pathway does not significantly affect the infection outcome.

### The autophagic flux is not necessary for infection

**Fig 6:**
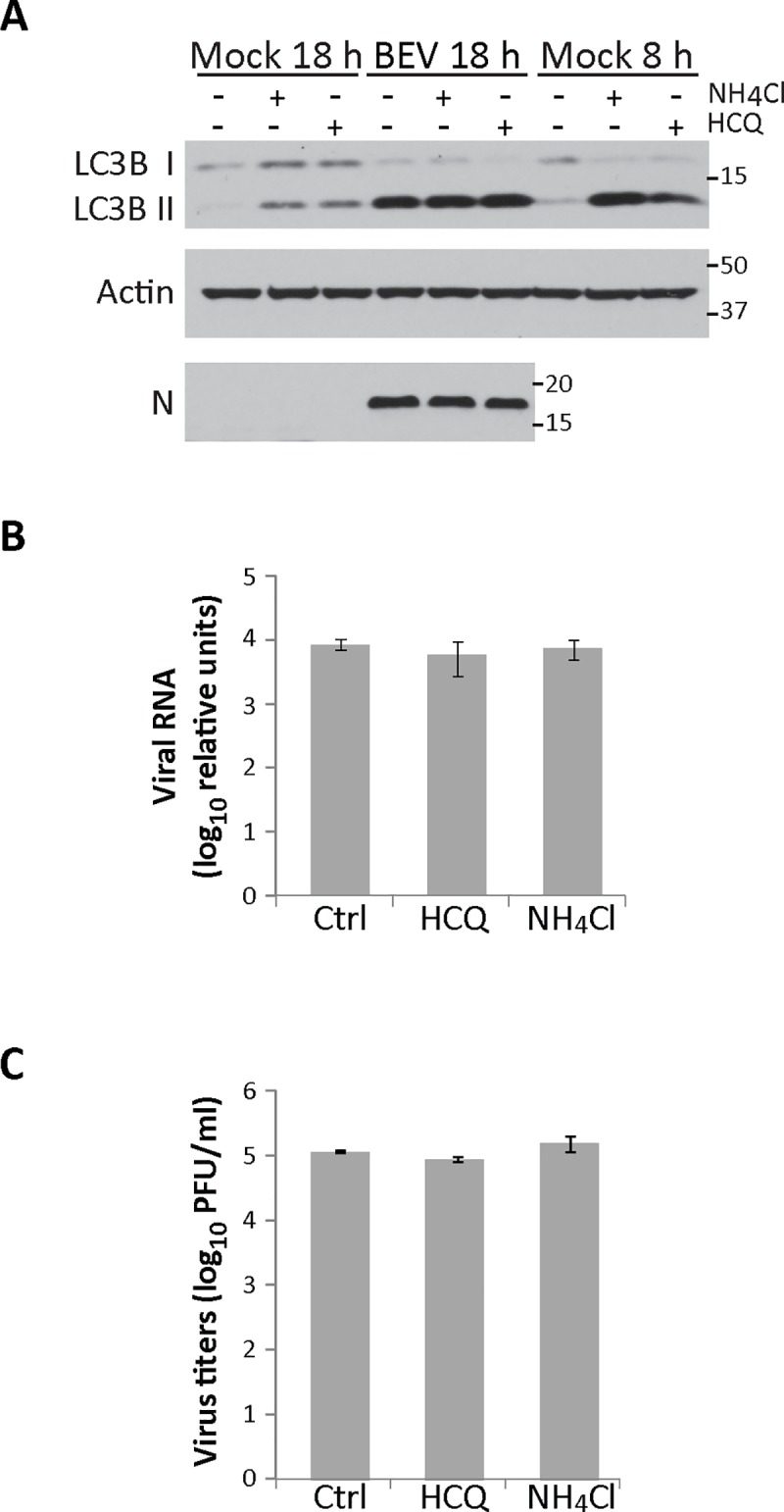
Effect of autophagy flux blockade during the early phase of BEV replication. E. Derm cells mock-infected or infected with BEV were treated from 3 to 8 h pi with NH_4_CL (20 mM), HCQ (17 μM) or left untreated (Control). After the treatment, cells were maintained in regular medium until the 18 h pi. Additionally, sets of mock-infected cells were harvested immediately after the 8 h treatments to test their efficacy. **A)** Cellular extracts were analyzed by Western blotting using specific antibodies against LC3B or BEV N. Actin was used as protein loading control. **B)** The amount of viral RNA in cell extracts (n = 3) produced in (A) was analyzed by qRT-PCR using primers specific for the BEV N gene and the housekeeping HPRT1, respectively. **C)** Supernatants (n = 3) produced in (A) were collected at 18 h pi and used to determine extracellular viral production.

To determine whether the autophagic flux plays a beneficial role on BEV replication, we designed an assay where autophagosome degradation was inhibited during the initiation and the establishment of the BEV replication process [15]. For this, E. Derm cells, either mock-infected or infected with BEV, were treated with NH_4_CL or HCQ from 3 to 8 h pi. Cultures were subsequently maintained in regular culture medium until the end of the assay (18 h pi). Cell extracts were analyzed by Western blot using an anti-LC3B antibody (Fig 6A). As a control to assessing the correct inhibition of autophagosome degradation by NH_4_CL and HCQ, sets of mock-infected cells were collected after concluding the corresponding treatments at 8 h pi. In these cells, accumulation of LC3B II was clearly detected (Fig 6A, Mock 8h). Nonetheless, autophagosome degradation was restored in mock-infected cells harvested at the end of the experiment (Mock 18 h), observing a high reduction in the level of LC3B II with respect to treated cells collected at 8 h pi. However, in infected cells major changes in the level of LC3B II between treated and untreated cells were not observed. Significantly, there were also no differences in the accumulation of the N viral protein. In addition, we analyzed by RT-qPCR the amount of viral RNA under these conditions, and again, we found no significant differences in the level of viral RNA accumulated in treated and untreated cells (Fig 6B). Similar results were obtained when infected cell supernatants were collected at 18 h pi and used to quantify extracellular virus production (Fig 6C).

**Fig 7:**
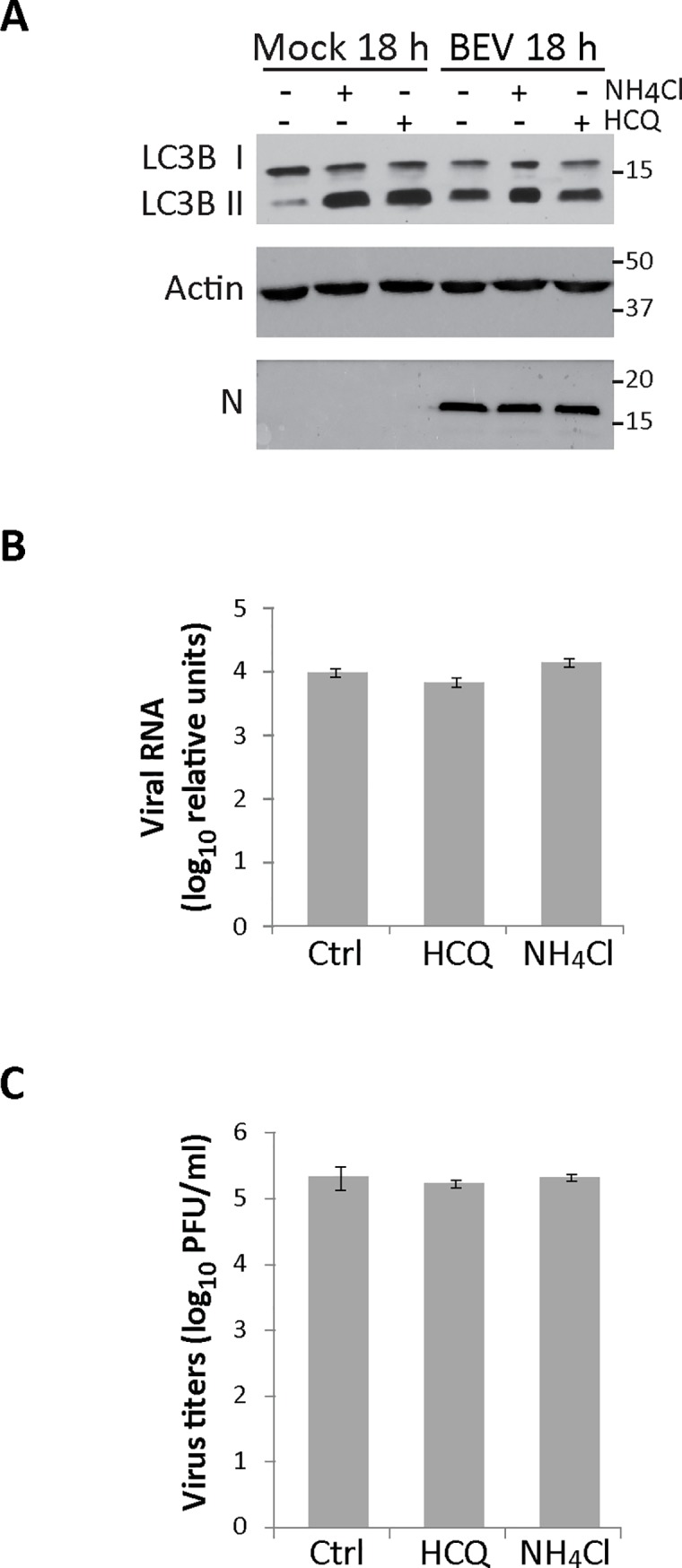
Effect of the autophagy flux blockade at late phase of the BEV replication cycle. Mock- or BEV-infected E. Derm cells were treated from 12 to 18 h pi with NH_4_CL (20 mM), HCQ (17 μM) or left untreated (Control). A) Cell extracts collected at 18 h pi were analyzed by Western blotting using antibodies against LC3B or the BEV N protein. Actin was used as protein loading control. B) The amount of viral RNA in cell extracts (n = 3) produced in (A) was analyzed by qRT-PCR using primers specific for BEV N gene and the housekeeping HPRT1, respectively. C) Cell supernatants (n = 3) produced in (A) were used to determine extracellular viral production.

Our results clearly indicate that the autophagic flux does not play a role on BEV replication. However, we could not rule out a possible effect at late times pi, when BEV infection induces an active autophagic flux in the cells. In the case of poliovirus, autophagy plays a beneficial role during infection, that is related with the maturation of poliovirus particles in acidic vesicles [76]. With this in mind, we treated mock- and BEV-infected E. Derm cells with NH_4_CL o HCQ from 12 to 18 h pi, thus blocking autophagosome degradation during the period of maximal extracellular BEV production. As shown in the Fig 7A, autophagosome degradation was inhibited in the treated cells, showing a high accumulation of LC3B II protein compared with untreated mock-infected cells. In cells infected and treated under these conditions, where the autophagy flux was impaired, no significant changes in the accumulation of neither the viral N protein (Fig 7A) nor viral RNA (Fig 7B) were detected. Similarly, the autophagic flux blockade did not affect the production of infectious extracellular viral particles (Fig 7C). An identical experiment was also carried out in MRC5 cells. In this case, extracts were analyzed by Western blotting using antibodies against p62, LC3 and the viral N protein, and the corresponding cell supernatants used for virus titration. Again, blocking the autophagic flux late during infection did not significantly affect BEV N protein accumulation (S2A Fig) or extracellular virus production (S2B Fig). In this experiment, we also analyzed the relative amount of p62 (S2A Fig). The amount of p62 in untreated BEV-infected cells was significantly reduced as compared with mock-infected cells (about 80% reduction). Similar results were gathered when comparing infected cells treated with autophagosome degradation inhibitors vs mock-infected cells treated with the same inhibitors (55% reduction for cells treated with NH_4_Cl and 72% for cells treated with HCQ) (S2A Fig).

Taken together, our results show that the autophagy pathway is activated in BEV infected cells late during the replication process, but this activation is irrelevant for the BEV life cycle.

### BEV could induce a selective autophagy

**Fig 8:**
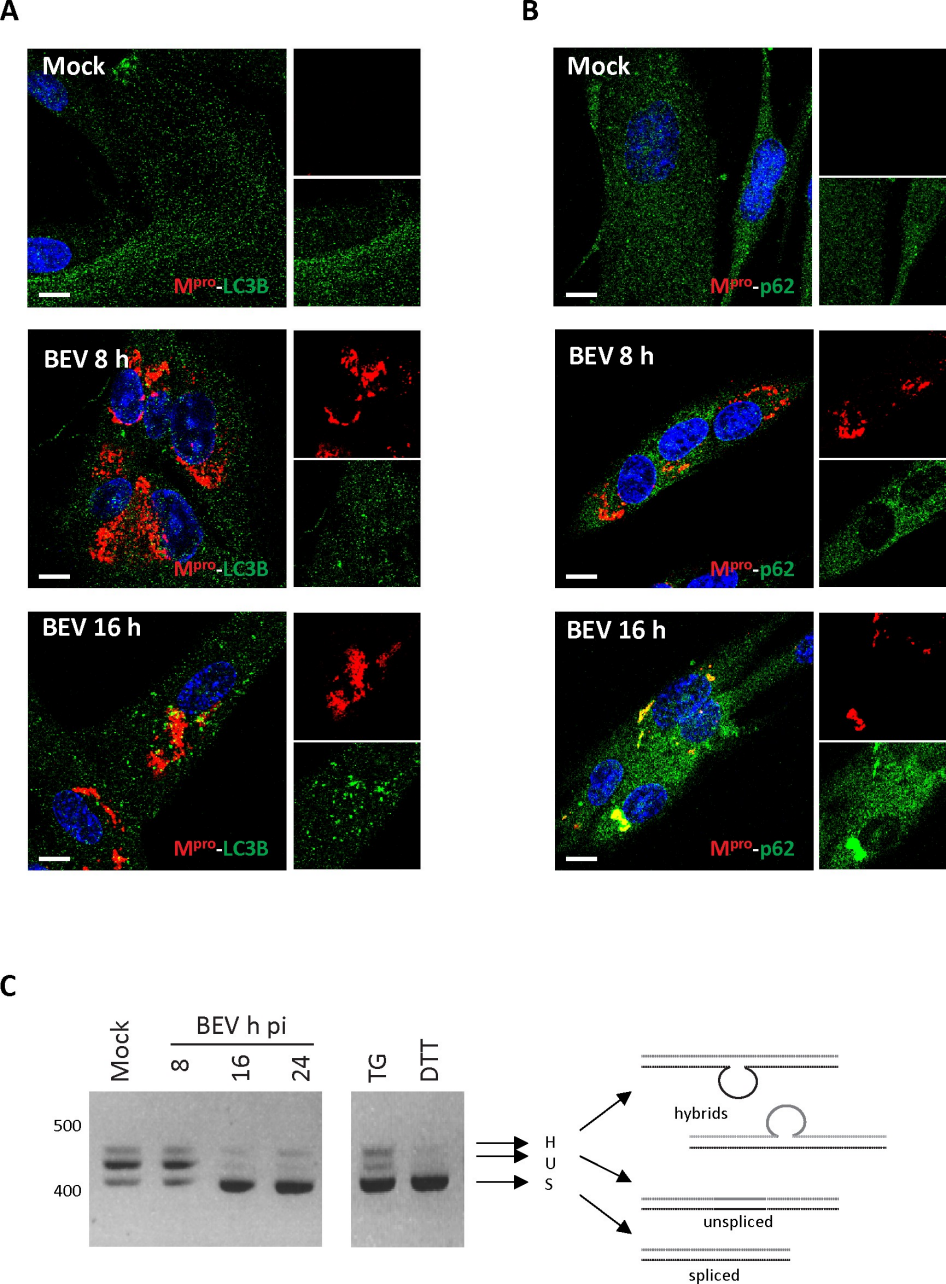
BEV induces selective autophagy and ER stress. **A-B)** Immunofluorescence analysis of mock- and BEV-infected MRC5 cells fixed at 8 and 16 h pi. Cells were immunolabeled with a specific antibody against the protease M^pro^ (red) of BEV and either a monoclonal antibody against LC3B (green) **(A)** or the polyclonal antibody against p62 (green) **(B)**. Cell nuclei were stained with DAPI (blue). Each panel shows a general, low magnification image of cell cultures, and, at right hand sides, enlarged representative areas exclusively showing the fluorescence corresponding to BEV M^pro^ (upper panels) and the cellular marker LC3 or the cargo protein p62 (lower panels). Scale bars, 10 μm. **C)** RNA from mock- or BEV-infected E. Derm cells was extracted at the indicated times pi, and used to analyze the splicing of XBP-1 mRNA by RT-PCR. As controls, RNA from mock-infected E. Derm cells treated with thapsigargin (200 nM, TG) or dithiothreitol (2 mM, DTT) were included. DNA amplicons were subjected to 2% agarose gel electrophoresis and visualized after ethidium bromide staining.

Traditionally, the autophagy pathway has been described as a non-selective bulk degradation process. However, in the last years this process has been described as highly selective, where the p62 protein, among others, acts as cargo receptor for the autophagic degradation of misfolded proteins or aggregates [77]. p62 harbors: i) a PB1 domain known to mediate self-oligomerization, and hence responsible for inducing the formation of aggregates; ii) an ubiquitin-binding association (UBA) domain; and iii) a LC3-interaction region (LIR), that provides the selective charge of ubiquitinated substrates to the autophagosome [57, 78, 79]. To check whether BEV induces selective autophagy, BEV-infected cells were processed for immunofluorescence using an anti-p62 antibody. Since, as mentioned above, none of the tested anti-p62 antibodies recognize the equine protein, assays were performed using human MRC5 cells. In order to visualize autophagosome formation, mock- or BEV-infected MRC5 cells were fixed at 8 and 16 h pi and processed for immunofluorescence using a monoclonal antibody against human LC3B and the anti-M^pro^ antibody to monitor virus infection. As shown in Fig 8A, LC3B dots accumulated at 16 h pi, and a high proportion of them were located surrounding the M^pro^ signal, as it is also observed during infection in E. Derm cells (S3 Fig). Next, we used the anti-p62 antibody. As shown in Fig 8B, the p62 protein exhibits a diffuse cytoplasmic localization in both mock-infected cells and cells infected for 8 h. In contrast, at 16 h pi p62 was aggregated within the region where the BEV M^pro^ localizes. Colocalization of p62 and M^pro^ proteins at 16 h pi was more evident in cells exhibiting compacted M^pro^ aggregates, indicating an advanced state of infection. These results suggest that BEV might induce selective autophagy in order to remove the excess of BEV proteins accumulated at late times pi.

It is well known that under stress conditions, eukaryotic cells maintain ER homeostasis by promoting the degradation of misfolded or toxic proteins using the ubiquitin proteasome system through the ER-associated protein degradation (ERAD) pathway [80, 81]. In addition, accumulating evidence indicates that autophagy is activated as a survival mechanism in cells subjected to ER stress. To determine whether BEV induces ER stress, we tested the induction of spliced X-box binding protein 1 (Xbp1), a transcription factor activated during ER stress, critically involved in the unfolded protein response (UPR). Xbp1 mRNA is spliced by the inositol-requiring enzyme 1 (IRE1) in response to ER stress, thus rendering the active transcription factor [82]. For this, RNA samples from mock- or BEV-infected E. Derm cells, collected at 8, 16 and 24 h pi were analyzed by RT-PCR with specific Xbp-1 primers [64]. In addition, RNA from cells treated during 20 min with ER stress-inducing chemicals thapsigargin (TG; 200 nM) or dithiothreitol (DTT; 2 mM) were included in the assay as controls. As shown in Fig 8C, mock-infected cells mainly contain the unspliced Xbp-1 mRNA (Xbp-1_U_) form, although a small amount of the spliced one was also detected (Xbp-1_S_). However, BEV infection caused a significant increase, akin to that observed in the cells treated with DTT or TG, in the relative amount of the spliced form at 16 and 24 h pi. Clearly, these results suggest that BEV induces ER stress at late time pi. Although a more comprehensive analysis is necessary, we believe that the excess of viral proteins accumulated at BEV late pi times may be instrumental in provoking ER stress, and thereby, in an effort to mitigate it, cells trigger the selective autophagy response.

## Discussion

The autophagy pathway is an essential component in the defense against infectious agents by promoting degradation of intracellular pathogens, as well as, by modulating innate and adaptive immune responses [37, 39]. However, some viruses have developed strategies to inhibit or even subvert the autophagy machinery for their benefit [65, 66]. In this regard, the relationship between torovirus infection and autophagy had not been investigated as yet. In the present work, we show using different approaches that the autophagy pathway is activated in BEV infected cells late during the infection cycle. Specifically, our results show a significant increase in the ratio between the lipidated LC3B (LC3B II) and its cytosolic form (LC3B I) at 16 and 24 h after infection with BEV. Consistently, in BEV-infected cells the reporter GFP-LC3 fusion protein is redistributed from a diffuse cytoplasmic localization (LC3B I) to a characteristic punctate pattern in autophagy vesicles (LC3B II) late during the infection. The same effect was observed when the endogenous LC3B protein was analyzed by immunofluorescence. Furthermore, our results using UV-inactivated BEV evidence that a replication-competent virus is required to induce autophagosome accumulation. Nevertheless, the accumulation of autophagic vacuoles can be due to both: i) autophagy induction; or ii) inhibition of the autophagy flux through autophagosome degradation. Our results, obtained by a well-established assay based on the use of the mCherry-EGFP-LC3B plasmid [57], together with the analysis of p62 turnover [67], unambiguously show that the autophagic flux remains active in BEV-infected cells, thus indicating that BEV infection activates the autophagy pathway.

Interestingly, neither autophagy induction by nutrient deprivation (EBSS), nor its pharmacological modulation with agents blocking autophagosome formation (3-MA or Wn) or autophagosome degradation (HCQ or NH_4_CL), significantly alter BEV replication. Similar results were obtained after infection of autophagy deficient cells lacking Beclin 1 or LC3B. Furthermore, autophagic flux inhibition at any point of the BEV replication cycle does not significantly affect the production of infectious extracellular virus. Overall, our results indicate that the autophagy pathway is activated in BEV infected cells at late time pi. However, this activation does not contribute to the final outcome of the infection, at least in the tested *in vitro* systems. Indeed, it is well established that autophagy modulates both innate and adaptive immune responses [36, 38, 83]. Consequently, we cannot rule out a possible impact of this pathway in BEV-infected animals at this point.

Like toroviruses, related members of the *Nidovirales* order are known to induce autophagy. However, the role of this pathway in nidovirus infections remains controversial [45–55]. Nidovirus replication occurs at the cell cytoplasm in association with DMVs [15, 29–34, 84–86]. From a structural point of view, DMVs are highly reminiscent of autophagosomes. This, together with the lack of accurate information about the origin of DMV membranes, has led to the suggestion that autophagosomes could provide the platform for nidovirus replication. Initial studies showed colocalization between membrane-bound replication-transcription complexes (RTC) of some coronaviruses and LC3 [45]. It was also reported that the replication of mouse hepatitis virus (MHV) was affected in autophagy deficient cells lacking the autophagy-related protein 5 (Atg5). Subsequent studies demonstrated that the porcine reproductive and respiratory syndrome virus (PRRSV) arterivirus also induces autophagy to sustain virus replication [49–51]. However, other studies contradict these findings, showing that Atg5 is not required for MHV [46], severe acute respiratory syndrome (SARS-CoV) [52] or avian infectious bronchitis virus (IBV) replication [87, 88]. Moreover, Snijder and coworkers did not find evidence for the colocalization of LC3 or GFP-LC3 with SARS-CoV RTCs [31]. Regarding this, although a large fraction of LC3-specific dots was found surrounding the BEV M^pro^ signal at late pi times, we did not observe colocalization of this protein and the LC3 polypeptide at any time of infection (Fig 8A and S3 Fig).

Some authors have suggested an autophagy-independent role for LC3 in both MHV [47] and EAV infections [53]. Surprisingly, these studies showed colocalization between DMV components and the LC3 I protein in autophagy-deficient cells lacking the autophagy-related protein 7 (Atg7), where viral replication was not affected. However, although autophagy was not strictly required, LC3 depletion markedly reduced the replication of these two viruses. Furthermore, it was also observed that MHV and EAV RTCs colocalize with some components of the ERAD machinery, i.e. EDEM1 and OS9. These findings combined with observation that EDEM1, OS9 and other ERAD factors are recycled in ER-derived vesicles coated with the non-lipidated LC3 I protein [89, 90], known as EDEMosomes, lead to suggestion that these vesicles may be the platform for nidovirus replication [47, 53]. Our attempts to determine whether the BEV RTCs colocalize with EDEM1 or OS9 were inconclusive. However, we have firmly established that BEV efficiently replicates in cells lacking LC3, thus ruling out a putative autophagy-independent role for LC3 in BEV infected cells.

### What is the role of autophagy in BEV infected cells?

An overall examination of our results leads us to rule out a proviral role of autophagy in BEV infected cells, since the different approaches used to modulate autophagy do not produce any beneficial changes on the BEV life cycle. On the contrary, we postulate that autophagy might act as an antiviral defense mechanism triggered by host cells to combat BEV infection. However, this mechanism is activated at late time pi, when the bulk of the virus progeny has already been released [15, 91], thus having little or no effect on the infection outcome.

As described above, cumulative information indicates that autophagy is a highly selective process. It is well documented that, among other proteins, p62 acts as cargo receptor for the autophagic degradation of aggregated proteins and damaged organelles, as well as for the removal of intracellular parasites [57, 78, 92]. The presence of p62 protein aggregates surrounded by the LC3 protein in close proximity to BEV M^pro^ was routinely found. However, direct colocalization between M^pro^ and LC3 was not clearly detected. Moreover, these aggregates were more apparent in cells with an advanced stage of infection. As mentioned above, the PB1 domain of p62 is involved in both self-oligomerization and aggregate formation. We hypothesize that p62 forms large aggregates with M^pro^, and only p62 molecules exposed on the surface of the aggregates could interact with LC3 thought its LIR domain. Although an in depth study is as yet required, this observation might indicate that BEV replication triggers a selective autophagy process at late time pi, where viral proteins are the substrate to be degraded. In line with this view, we also observed that BEV induces ER stress at the time when the autophagy is happening. Therefore, the accumulation of viral proteins at late times pi may trigger ER stress, and as consequence of this, cells activate selective autophagy to alleviate it. It is well known that cells respond to the ER stress through the activation of the ERAD pathway, which under stress conditions leads to ubiquitination of misfolded proteins to be degraded by the proteasome [80]. However, an excessive accumulation of misfolded proteins can saturate the proteasome. Under these circumstances, cells respond by removing ubiquitinated substrates via selective autophagy [78, 79]. Nevertheless, future studies would be necessary to precisely determine the role of p62 protein during the BEV replication process.

In a previous study from our group, we observed that BEV also induces apoptosis by the activation of PKR and the phosphorylation of translation initiation factor 2-alpha (eIF2-α) at the very end of the replication process, once the viral progeny is produced [56]. It seems clear that both, autophagy and apoptosis are antiviral defense mechanisms. Overall, our data suggest that the massive accumulation of viral proteins during the last stages of the BEV replicative process may be the primary cause of the ER stress and thereby, in an effort to mitigate this stress, cells respond activating the autophagy pathway. However, as it has been shown to occur in other coronaviruses [93], the incapacity to ameliorate the stress might lead to cell death by apoptosis.

## Supporting information

S1 Fig Effect of Beclin 1 knockdown on BEV replication. E. Derm cells were transduced with retroviral vectors expressing either a short harping RNA against Beclin 1 (shRNA Beclin 1-KD) or an irrelevant shRNA (shRNA Control). **A)** Extracts from cells expressing Beclin 1 or Control shRNA were collected at 16 h pi and analyzed by Western blotting using the anti-Beclin 1 antibody. Total proteins were stained with Ponceau solution as loading control. **B)** Supernatants of E. Derm cells expressing shRNA Beclin 1 or shRNA Control infected with BEV (n = 3) were used to determine the extracellular virus yields.
(EPS)

S2 Fig Effect of the autophagy flux blockade induced late during the BEV replication process. Mock- or BEV-infected MRC5 cells were treated from 12–18 h pi with NH_4_CL (20 mM), HCQ (17μM) or left untreated (Control). **A)** Cell extracts collected at 18 h pi were analyzed by Western blot with antibodies against LC3B, p62 or the BEV N protein. Actin was used as protein loading control. **B)** Cell supernatants (n = 3) produced in (A) were used to determine the extracellular viral production.
(EPS)

S3 Fig Immunofluorescence analysis of LC3 and M^pro^ proteins in BEV-infected E. Derm cells. Mock-infected or BEV-infected cells were fixed 16 h pi and processed for immunofluorescence with antibodies specific for the LC3B (green) and M^pro^ (red). Nuclei were stained with DAPI. Scale bars, 10 μm.
(EPS)
